# In the national epidemiological bulletins – a selection from recent issues

**DOI:** 10.2807/1560-7917.ES.2019.24.13.1903281

**Published:** 2019-03-28

**Authors:** Eurosurveillance editorial team

**Affiliations:** 1 European Centre for Disease Prevention and Control (ECDC), Stockholm, Sweden

**Keywords:** infectious diseases, public health, Europe

**Healthcare-associated infections and antibiotic resistance**

 

Clostridium difficile infection in Ireland, 2017

Epi-Insight 2019;20(1)

January 2019, Ireland

http://ndsc.newsweaver.ie/epiinsight/1scooyoz373n0ykqgxo0gg?a=1&p=54394726&t=17517774

 

**Food- and waterborne diseases**

 

Routine reports of gastrointestinal infections in humans, England and Wales: January and February, 2019

Health Protection Report; 13(9)

15 March 2019, United Kingdom

https://assets.publishing.service.gov.uk/government/uploads/system/uploads/attachment_data/file/786477/hpr0919_GIs.pdf

 

**Hepatitides**

 

Laboratory reports of hepatitis A and C in England and Wales: July to September 2018

Health Protection Report; 13(3)

25 January 2019, United Kingdom

https://assets.publishing.service.gov.uk/government/uploads/system/uploads/attachment_data/file/773602/hpr0319_hepAC.pdf

 

**Vaccine-preventable diseases**

 

Diphtheria in England: 2018

Health Protection Report; 13(10)

22 March 2019, United Kingdom

https://assets.publishing.service.gov.uk/government/uploads/system/uploads/attachment_data/file/788746/hpr1019_dphthr.pdf

 

Tetanus in England: 2018

Health Protection Report; 13(10)

22 March 2019, United Kingdom

https://assets.publishing.service.gov.uk/government/uploads/system/uploads/attachment_data/file/788744/hpr1019_ttns.pdf

 

Pertussis - vaccinating mum is good for baby – update on antenatal pertussis vaccination

Epi-Insight 2019;20(3)

March 2019, Ireland

http://ndsc.newsweaver.ie/epiinsight/zvpnkwflilyn0ykqgxo0gg?a=1&p=54630557&t=17517774

 

The need to achieve high vaccine coverage to sustain rubella elimination in Ireland

Epi-Insight 2019;20(3)

March 2019, Ireland

http://ndsc.newsweaver.ie/epiinsight/dgoyhvql9ntn0ykqgxo0gg?a=1&p=54630561&t=17517774

 

Laboratory-confirmed cases of measles, rubella and mumps, England: October to December 2018

Health Protection Report; 13(8)

28 February 2019, United Kingdom

https://assets.publishing.service.gov.uk/government/uploads/system/uploads/attachment_data/file/782191/hpr0819_mmr.pdf

 

Laboratory confirmed cases of invasive meningococcal infection (England): October to December 2018

Health Protection Report; 13(7)

22 February 2019, United Kingdom

https://assets.publishing.service.gov.uk/government/uploads/system/uploads/attachment_data/file/780984/hpr0719_imd.pdf

 

Laboratory reports of *Haemophilus influenzae* by age group and serotype (England): 2018

Health Protection Report; 13(7)

22 February 2019, United Kingdom

https://assets.publishing.service.gov.uk/government/uploads/system/uploads/attachment_data/file/780976/hpr0719_hmphls-nflnz.pdf

 

Pertussis vaccination programme for pregnant women update: vaccine coverage in England, July to September 2018

Health Protection Report; 13(7)

22 February 2019, United Kingdom

https://assets.publishing.service.gov.uk/government/uploads/system/uploads/attachment_data/file/780991/hpr0719_prntl-prtsss-vc.pdf

 

Trends in invasive meningococcal disease in Ireland, 1999-2019

Epi-Insight 2019;20(2)

February 2019, Ireland

http://ndsc.newsweaver.ie/epiinsight/tyzbecfnxf6n0ykqgxo0gg?email=true&a=1&p=54506595&t=17517774

 

**Respiratory diseases**

 

Outbreak of tuberculosis in a school in Dresden, 2017−2018

Epidemiologisches Bulletin 11-12/2019

14 March 2019, Germany

https://www.rki.de/DE/Content/Infekt/EpidBull/Archiv/2019/Ausgaben/11_12_19.pdf?__blob=publicationFile

 

Probable case of reinfection with *Legionella pneumophila*

Epidemiologisches Bulletin 1/2019

3 January 2019, Germany

https://www.rki.de/DE/Content/Infekt/EpidBull/Archiv/2019/Ausgaben/01_19.pdf?__blob=publicationFile

 

**Zoonoses and vector-borne diseases**

 

Common animal associated infections quarterly report (England and Wales): fourth quarter 2018

Health Protection Report; 13(9)

15 March 2019, United Kingdom

https://assets.publishing.service.gov.uk/government/uploads/system/uploads/attachment_data/file/786463/hpr0919_zoos.pdf

 

Case of chronic Q fever

Vlaams infectieziektebulletin 2019(1)

March 2019, Belgium

https://www.zorg-en-gezondheid.be/sites/default/files/atoms/files/VIB%202019-1%20-%20DEF.pdf

 

Historical aspects of Q fever

Vlaams infectieziektebulletin 2019(1)

March 2019, Belgium

https://www.zorg-en-gezondheid.be/sites/default/files/atoms/files/VIB%202019-1%20-%20DEF.pdf

 

Creutzfeldt-Jakob disease (CJD) biannual update (February 2019)

Health Protection Report; 13(6)

15 February 2019, United Kingdom

https://assets.publishing.service.gov.uk/government/uploads/system/uploads/attachment_data/file/779110/hpr0619_cjd.pdf

 

TBE: risk areas in Germany (as of January 2019)

Epidemiologisches Bulletin 7/2019

14 February 2019, Germany

https://www.rki.de/DE/Content/Infekt/EpidBull/Archiv/2019/Ausgaben/07_19.pdf?__blob=publicationFile

 

Leptospirosis 2017-2018

EPI-NEWS 4/5, 2019

6 February 2019, Denmark

https://en.ssi.dk/news/epi-news/2019/no-4-5---2019

 

**Other**

 

Pregnancy and infectious diseases in Ireland

Epi-Insight 2019;20(3)

March 2019, Ireland

http://ndsc.newsweaver.ie/epiinsight/d95m2dq9kepn0ykqgxo0gg?email=true&a=2&p=54630552&t=17517804

 

Group A streptococcal infections: first report of seasonal activity, 2018/19

Health Protection Report; 13(8)

28 February 2019, United Kingdom

https://assets.publishing.service.gov.uk/government/uploads/system/uploads/attachment_data/file/782182/hpr0819_sf-gas.pdf

